# COMPARING THE OUTCOMES OF VIRTUAL VERSUS IN-PERSON DELIVERY OF AN EVIDENCE-BASED FALLS PREVENTION PROGRAM

**DOI:** 10.1093/geroni/igad104.2282

**Published:** 2023-12-21

**Authors:** Tamara Herrick, Kirsten Dorsey, Sarah Hallen, Heidi Wierman

**Affiliations:** MaineHealth, Portland, Maine, United States; MaineHealth, Portland, Maine, United States; MaineHealth, Portland, Maine, United States; MaineHealth- Maine Medical Partners, Portland, Maine, United States

## Abstract

Each year one in four older adults has a fall1 and only about half tell their doctor or healthcare provider2. One way to prevent falls is to provide older adults with the evidence-based tools to prevent them. A Matter of Balance: Managing Concerns about Falls (MOB), is a top tier program for grant funding from the Administration for Community Living Falls Prevention Program. MOB has been shown to reduce the fear of falling and increase activity levels among older adults through cognitive restructuring and balance exercises.8 Prior to 2020, MOB was only offered as an in-person, small group (8-12 participants), community based intervention over 8 two-hour sessions led by 2 volunteer facilitators. In response to the global COVID-19 pandemic, MaineHealth’s Partnership for Healthy Aging adapted the Lay Leader model of MOB for virtual delivery (MOB-V). Review of MOB-V pilot data suggested that it was also effective in reducing the fear of falling and increasing the activity levels of older adults. Based on this success, the MOB-V was more widely disseminated during 2021 and 2022, but to date no comprehensive program evaluation of the MOB-V has been completed. We will present the preliminary results of our evaluation of participant data collected from the National Falls Prevention database between 2021 through 2022.

